# cAMP/PKA-CREB-BDNF signaling pathway in hippocampus mediates cyclooxygenase 2-induced learning/memory deficits of rats subjected to chronic unpredictable mild stress

**DOI:** 10.18632/oncotarget.16009

**Published:** 2017-03-08

**Authors:** Ying Luo, Shengnan Kuang, Huan Li, Dongzhi Ran, Junqing Yang

**Affiliations:** ^1^ Department of Pharmacology, Chongqing Medical University, Chongqing, China; ^2^ Chongqing Key Laboratory of Biochemistry and Molecular Pharmacology, Chongqing, China; ^3^ Department of Pharmacy, People's Hospital of Rongchang, Chongqing, China

**Keywords:** cyclooxygenase2, inflammation, cognitive impairment, hippocampus, BDNF, Neuroscience

## Abstract

To investigate the mechanism of cyclooxygenase 2 (COX2) in learning and memory impairments in rats subjected to chronic unpredictable mild stress (CUMS), meloxicam was used intragastrically to inhibit the activity of cyclooxygenase 2. Moreover, cyclooxygenase 2 over-expressing or RNA interfere lentivirus was injected intraventricularly to increase or decrease the enzyme's expression, respectively. The body weights and sucrose consumption were used to analyze depressive behaviors, while the Morris water maze and step-down-type passive avoidance tests were carried out to evaluate the learning-memory functions. The levels of inflammatory cytokines were measured to estimate inflammation and the contents of cyclic adenosine monophosphate (cAMP) were used to measure the levels of the second messenger. Changes in cyclooxygenase 2 mRNA levels were analyzed using reverse transcription polymerase chain reaction. Moreover, the expression of cyclooxygenase 2, brain-derived neurotrophic factor (BDNF), prostaglandins receptor 3 (EP3), protein kinase A (PKA), cAMP response element binding protein (CREB), and phosphorylated CREB were estimated using immunohistochemical staining or western blotting. The results showed that CUMS led to significant depressive-like behaviors and learning and memory dysfunctions. Also, the cAMP levels decreased significantly, while levels of inflammatory cytokines and prostaglandins E2 increased significantly. The expressions of PKA, BDNF, phosphorylated CREB/CREB declined and cyclooxygenase 2 was increased. Meloxicam and cyclooxygenase 2 RNA interfere lentivirus reversed the changes caused by CUMS while cyclooxygenase 2-overexpressing lentivirus worsened these abnormalities. The findings also showed that CUMS increased cyclooxygenase 2 expression, which can cause learning and memory impairments, mainly through activating the hippocampal neuronal cAMP/PKA-CREB-BDNF signaling pathways.

## INTRODUCTION

Depression is a public health threat, ranking third among the leading causes of global disease burden [1]. The mood disorders of depression are often accompanied by cognitive symptoms, such as deficits in learning and memory, difficulty in decisions making, and loss of cognitive flexibility [2, 3]. The increasing evidence indicates that these cognitive deficiencies may be an early episode in depression, and may hence predict the likelihood of recovery, however, its pathophysiological basis remains poorly understood [4, 5].

Over the last decades, increasing evidence has led to the hypothesis that inflammatory processes are involved in the pathophysiology of depression [6]. In experiments among healthy volunteers, injecting typhoid, but not placebo, have yielded an inflammatory response that was indicated by greater circulating interleukin-6 and mood reduction [7]. Increased mean plasma levels of pro-inflammatory cytokines such as the tumor necrosis factor alpha (TNF-α), interleukin-1 (IL-1) and interleukin-6 (IL-6) have been reported in patients with clinical depression [8]. In nonhuman animal models [9], administration of high doses of interferon-α has been reported to induce depressive-like symptoms, which could be attenuated with repeated anti-depressant treatment. These pieces of evidence clearly indicate a crosstalk between chronic inflammation and depression. However, it remains unclear whether the cognitive dysfunction in depression is a consequence of neuroinflammation. Recent studies have reported a strong association between inflammation and deficits in learning and memory in animal models for neurodegenerative diseases including Parkinson's disease, Alzheimer's disease (AD) and amyotrophic lateral sclerosis [10–13]. The possibility of an association between inflammation and deficits in learning and memory in neurodegenerative diseases gives rise to the possible existence of this association in depression as well. In this case, it remains to be investigated, whether pharmacological suppression of inflammation is effective in preventing learning and memory impairments in depression.

Non-steroidal anti-inflammatory drugs (NSAIDs) are one of the most commonly used drugs in medicine. These drugs are agents that may be capable of interrupting the neurotoxic cascade in via the inhibition of the cyclooxygenase activity [14]. cyclooxygenase is a lipid-peroxidizing enzyme that is involved in the conversion of arachidonic acid into prostaglandins. Cyclooxygenase can be sorted into two forms, the constitutive cyclooxygenase 1 (COX1) and the inducible cyclooxygenase 2 (COX2). COX1 is constitutively expressed in several human tissues, whereas COX2 is primarily considered as the inducible form of the enzyme [15]. It has been confirmed that COX2 mRNA and protein levels are elevated in neurons in AD patients [16, 17]. In animal models, administration of ibuprofen has been reported to decrease the cerebral prostaglandin E2 (PGE2) levels and to improve the depressive-like behavior through the inhibition of COX2 [7]. These studies have implied a possible role of COX2 in the cognitive deficits observed in depression. However, the link between COX2 and cognitive dysfunction induced by depression remains unknown.

The hippocampus is the primary structure of the brain that has been confirmed to be involved in the process of learning and memory [18]. Altered synaptic plasticity of hippocampal neurons has been extensively demonstrated in cognitive and emotional disorders, with the brain-derived neurotrophic factor (BDNF) being the most important target associated with such observations. Indeed, in mature neurons, BDNF plays a pivotal role in synaptic plasticity, promoting neurotransmission, and regulating the receptors sensitivity [19]. In immature neurons, BDNF participates in their differentiation and maturation as well as in the regulation of neuronal plasticity [20]. In humans, BDNF Met polymorphism has been associated with short-term episodic memory impairments as well as increased susceptibility to neuropsychiatric disorders, such as affective disorders and schizophrenia. This may explain the common clinical symptom related to cognitive impairments [21, 22]. All these previous studies have suggested an involvement of both COX2 and BDNF in the pathogenesis of cognitive deficits. However, whether COX2 and BDNF share a common pathway in the pathophysiological processes underlying cognitive dysfunctions observed in depression remains unclear. The link between both is also unknown. In our previous study, we reported an elevated COX2 expression and a decreased BDNF content in the hippocampus of rats subjected to chronic unpredictable mild stress (CUMS) [23]. Therefore, in the present study we aim to explore the link among COX2, BDNF and cognitive deficits in CUMS-exposed rats. For this, we (1) administered meloxicam to inhibit the activity of COX2, and (2) administered COX2-silencer lentiviral vectors (LV-si-COX2) or COX2 over-expressing lentivirus via intracerebroventricular injection to change the expression status of COX2 in rats subjected to CUMS. We further analyzed the changes of the COX2 downstream signaling pathways, neuronal plasticity, and learning and memory processes. Our results revealed a previously unknown signaling pathway that links COX2 with learning and memory deficits induced by CUMS exposure. This finding proposes a novel therapeutic intervention to treat cognitive dysfunctions observed in depression.

## RESULTS

### Inhibited activity of COX2 by meloxicam improved symptoms of depression induced by CUMS

The body weights and sucrose consumption of all subgroups are shown in Figure 2a and Figure 2b, respectively. Body weight and sucrose intake in each of the subgroups was not significantly different at the beginning of the CUMS procedure. Six weeks following the CUMS treatment, a significant decrease in body weight and sucrose intake was observed in CUMS groups (compared with the control naive group, *P* < 0.01 and *P* < 0.01, respectively). The results showed that administration of meloxicam (1 or 3 mg/kg), sertraline (5 mg/kg) significantly increased the body weight (*P* < 0.01, *P* < 0.01 and *P* < 0.05) and sucrose consumption (*P* < 0.01, *P* < 0.01 and *P* < 0.01) compared with CUMS group.

**Figure 1:**
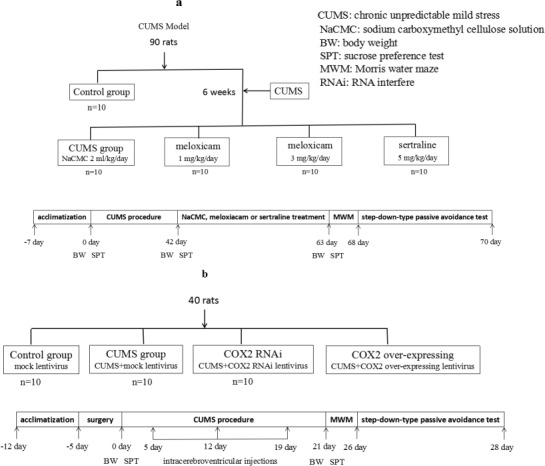
Experimental procedures Animal groups, treatments and abbreviations in figure.

**Figure 2:**
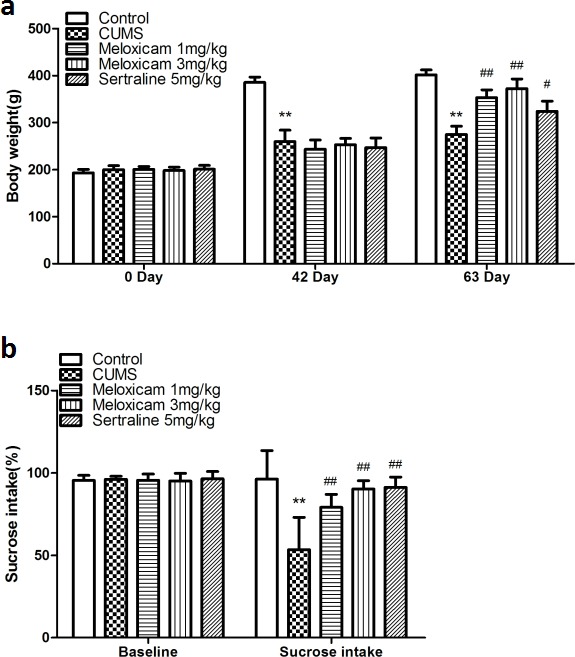
Changes in depressive-like behaviors induced by CUMS and effect of meloxicam treatment **a**. Changes of body weight in CUMS rats after treatment with meloxicam(1 and 3mg/kg, i.g.), sertraline(5mg/kg, i.g.). **B**. The effects of meloxicam(1 and 3mg/kg, i.g.), sertraline (5mg/kg, i.g.) on the sucrose preference test in CUMS-treated rats. Each value represents the mean ± SD using a one-way ANOVA with a Bonferroni correction. **P < 0.01 vs control group. ^#^and ^##^ *P* < 0.05 and < 0.01 vs CUMS group. *n* = 10.

### Suppression of COX2 expression improved depressive symptoms induced by CUMS. Whereas, intraventricular injection of COX2 over-expressing lentivirus exaggerated the depressive behavior in CUMS-treated rats

**Figure 3:**
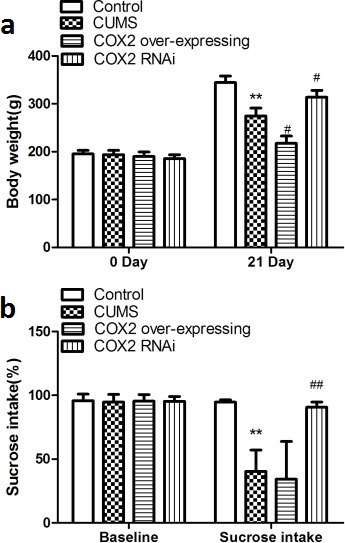
Effects of COX2 over-expression or RNAi on the changes of depressive-like behaviors in CUMS-treated rats **a.** Effects of COX2 over-expressing lentivirus, LV-si-COX2 or LV-Mock (15 ul, 10 ^8^ TU/ml, intracerebroventricular injection, respectively) on the changes of body weight in CUMS-treated rats. **b**. Effects of COX2 over-expressing lentivirus, LV-si-COX2 or LV-Mock (15 ul, 10 ^8^ TU/ml, intracerebroventricular injection, respectively) on the changes of sucrose preference test in CUMS-treated rats. Each value represents the mean ± SD using a one-way ANOVA with a Bonferroni correction. **P < 0.01 vs control group. ^#^ and ^##^ *P* < 0.05 and < 0.01 vs the CUMS group. *n* = 10.

As shown in the Figure 3a and Figure 3c, there was no significant difference among groups in body weight and sucrose intake among groups at the beginning. CUMS significantly reduced the body weight and sucrose performance in the LV-Mock-treated CUMS groups (compared with the control naive group, *P* < 0.01 and *P* < 0.01, respectively). Upon suppression of the COX2 expression by COX2 siRNA, this reduction in both body weight and sucrose intake was reversed (compared with the LV-Mock-treated CUMS groups, *P* < 0.05 and *P* < 0.01, respectively). Whereas, over-expression of the COX2 only facilitated the reduction in the weight body (compared with the LV-Mock-treated CUMS groups, *P* < 0.01).

### The inhibition of COX2 activity by meloxicam administration reduced CUMS-induced learning and memory impairment in rats

**Figure 4:**
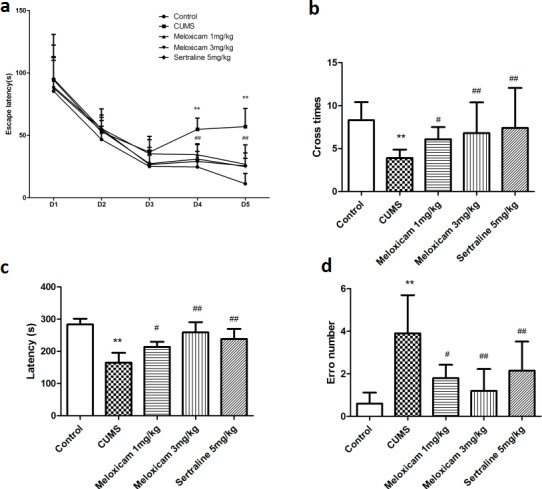
The effects of meloxicam treatment on learning and memory of CUMS-treated rats based on the MWM task and the step-down-type passive avoidance test **a.** The escape latency and number of platform crosses in the Morris water maze (MWM) test in CUMS rats after treatment with meloxicam (1 and 3mg/kg, i.g.), sertraline(5mg/kg, i.g.). **b**. The effects of meloxicam (1 and 3mg/kg, i.g.), sertraline(5mg/kg, i.g.) on the number of errors and latency in the step-down-type passive avoidance test in CUMS-treated rats. Each value represents the mean ± SD, datas for the lantency in MWM were assessed by repeated measures analysis of variance (ANOVA), datas for cross number in MWM and data in step-down-type passive avoidance test were measured by a one-way ANOVA with a Bonferroni correction. **P < 0.01 vs control group. ^#^ and ^##^ *P* < 0.05 and < 0.01 vs the CUMS group. *n* = 10.

The results obtained in spatial-learning acquisition and retention in the Morris water maze (MWM) test illustrated that all rat groups were able to learn over the trial days, although sodium carboxymethyl cellulose solution (CMC-Na)-treated CUMS group exhibited a slow learning progress measured as latency to escape from the platform on day 4 [F(2, 20) = 10.734, *P* = 0.001] and day 5 [F(2, 20) = 11.271, *P* = 0.001], and a decrease in the number of crosses in the MWM (*P* < 0.01) compared with naive group (Figure 4a, 4b). Figure 4 also shows the administration of meloxicam (1 or 3 mg/kg), sertraline (5 mg/kg) significantly decreased the latency to escape on day 4 [F(2, 20) = 5.748, *P* = 0.024, F(2, 20)= 8.9741 *P* = 0.0029, F(2, 20)= 8.784, *P* = 0.008, respectively] and day 5 [F(2, 20) = 6.297, *P* = 0.019, F(2, 20) = 9.2517, *P* = 0.003, F(2, 20) = 8.083, *P* = 0.005, respectively] and increased the number of crosses in the area where the platform previously existed in (*P* < 0.05, *P* < 0.01 and *P* < 0.01).

The step-down-type passive avoidance test (Figure 4c, 4d) was used to assess negative feedback learning. A one-way ANOVA revealed a significant effect of CUMS, indicating a decrease in latency (*P* < 0.01) and increase error number (*P* < 0.01) in CUMS group. However, treatment with meloxicam (1 or 3 mg/kg) or sertraline (5 mg/kg) showed significantly increase of latency (*P* < 0.05, *P* < 0.01 and *P* < 0.01) and decrease error number (*P* < 0.05, *P* < 0.01 and *P* < 0.01), as compared to the CUMS group, indicating better memory performance.

### Suppression of COX2 expression reduced CUMS-induced learning and memory impairment in rats, however, COX2 over-expressing lentivirus intraventricular injection aggravated the learning and memory impairment in CUMS-treated rats

**Figure 5:**
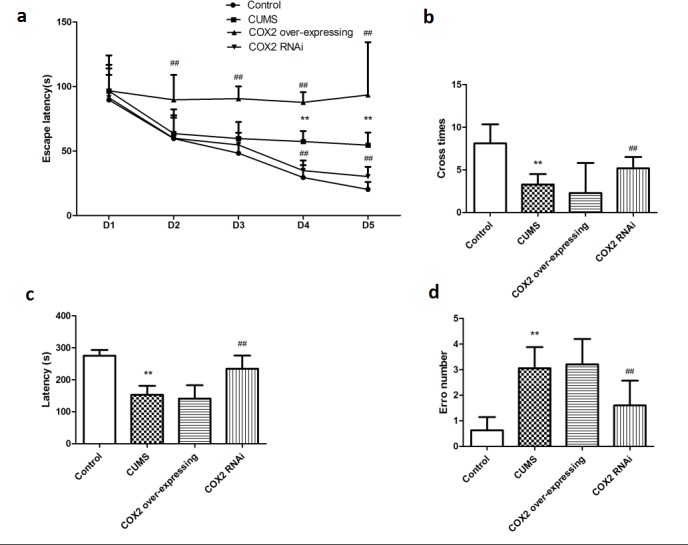
The effects of COX2 expression status on learning and memory of CUMS-treated rats based on the MWM task and the step-down-type passive avoidance test **a.** The escape latency and number of platform crosses in the Morris water maze (MWM) test in CUMS rats after treatment with COX2 over-expressing lentivirus, LV-si-COX2 or LV-Mock (15 ul, 10 ^8^ TU/ml, intracerebroventricular injection, respectively). **b**. The effects of COX2 over-expressing lentivirus, LV-si-COX2 or LV-Mock (15 ul, 10 ^8^ TU/ml, intracerebroventricular injection, respectively) on the number of errors and latency in the step-down-type passive avoidance test in CUMS-treated rats. Each value represents the mean ± SD, datas for the lantency in MWM were assessed by repeated measures analysis of variance (ANOVA), datas for cross number in MWM and data in step-down-type passive avoidance test were measured by a one-way ANOVA with a Bonferroni correction. **P < 0.01 vs control group. ^#^ and ^##^ *P* < 0.05 and < 0.01 vs the CUMS group. *n* = 10.

In a treatment of CUMS at the same time subjecting rats with LV-si-COX2 or COX2 over-expressing lentivirus and then assessed animals’ behavior in the MWM and step-down-type passive avoidance test. As shown in Figure 5a and 5b, CUMS prolonged the time spent seeking the platform and the escape latency in the MWM test in the LV-Mock-treated CUMS groups on day 4 and day 5 [F(2, 20) = 10.945, P = 0.001 and F(2, 20) = 10.427, *P* = 0.001, respectively], and induced a decrease in the number of platform crosses in the MWM (*P* < 0.01). CUMS decreased the latency to step down (*P* < 0.01) and elevated the number of errors (*P* < 0.01) in the step-down-type passive avoidance test (Figure 5c and 5d). Figure 5 also showed that intracerebroventricular injection of LV-si-COX2 significantly decreased escape latency on the 4th [F(2, 10) = 8.483, *P* = 0.005] and 5th [F(2, 10) = 8.013, *P* = 0.006] day and increased the number of platform crosses (*P* < 0.01) in the MWM. Suppression of the COX2 expression also prolonged the latency (*P* < 0.01) and decreased the number of errors (*P* < 0.01) in the step-down-type passive avoidance test compared to the LV-Mock-treated CUMS group. Conversely, COX2 over-express lentivirus intraventricular injection prolonged the latency on 2nd [F(2, 10) = 7.682, *P* = 0.008], 3rd [F(2, 10) = 7.762, *P* = 0.008], 4th [F(2, 10) = 8.081, *P* = 0.006] and 5th [F(2, 10) = 8.628, *P* = 0.004] day in MWM test compared to the LV-Mock-treated CUMS group, indicating increased severity of learning/memory dysfunction.

### The administration of meloxicam prevented the CUMS-induced elevation of inflammatory mediator levels in the hippocampus

**Figure 6:**
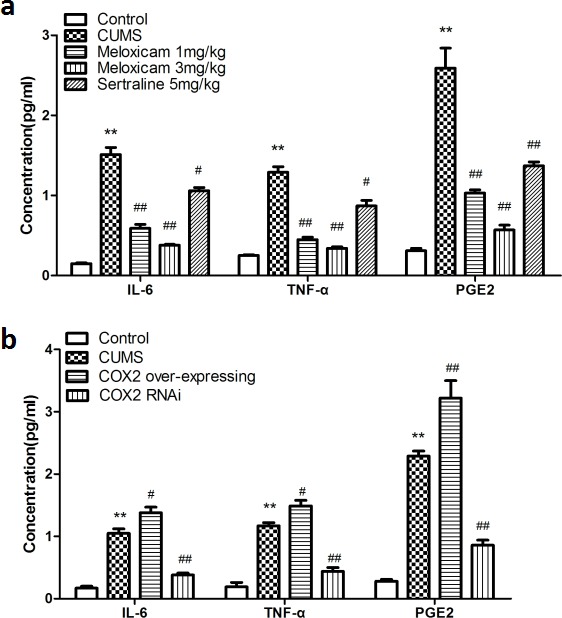
Changes of the concentration of inflammatory mediators in the hippocampus of CUMS rats upon inhibite the activity of COX2 or change the expression status of COX2 **a**. The concentration of IL-6, TNF-α, PGE2 in the hippocampus of CUMS-treated rats after treatments of meloxicam (1 and 3mg/kg, i.g.), sertraline(5mg/kg, i.g.). **b**. The effects of COX2 over-expressing lentivirus, LV-si-COX2 or LV-Mock (15 ul, 10 ^8^ TU/ml) intracerebroventricular injection on the levels of IL-6, TNF-α, PGE2 in the hippocampus of CUMS-treated rats. Each value represents the mean ± SD using a one-way ANOVA with a Bonferroni correction. **P < 0.01 vs control group. ^#^ and ^##^ *P* < 0.05 and < 0.01 vs the CUMS group. *n* = 10.

To understand the underlying mechanism how COX2 contributes cognitive deficits in the CUMS-treated rats, we assayed the levels of inflammatory biomarkers, including TNF-α, prostaglandins E2 (PGE2) and IL-6. As shown in Figure 6a, CUMS significantly increased the levels of IL-6 (*P* < 0.01), TNF-α (*P* < 0.01) and PGE2 (*P* < 0.01) compared with naive group. The administration of meloxicam (1 or 3 mg/kg) and sertraline (5 mg/kg) reduced the concentration of IL-6 (*P* < 0.01, *P* < 0.01 and *P* < 0.05 respectively), TNF-α (*P* < 0.01, *P* < 0.01 and *P* < 0.05 respectively) and PGE2 (*P* < 0.01, *P* < 0.01 and *P* < 0.01, respectively).

### Intracerebroventricular injection of LV-si-COX2 prevented the CUMS-induced elevation in levels of inflammatory mediators in the hippocampus. Whereas, COX2 overexpression caused an exacerbation of inflammation in the hippocampus

CUMS increased levels of IL-6 (*P* < 0.01), TNF-α (*P* < 0.01) and PGE2 (*P* < 0.01) significantly compared with naive group, suppression of the COX2 expression by LV-si-COX2 intracerebroventricular injection prevent the elevation of IL-6 (*P* < 0.01), TNF-α (*P* < 0.01) and PGE2 (*P* < 0.01); however, COX2 over-expression aggravated the increase of IL-6 (*P* < 0.05), TNF-α (*P* < 0.05) and PGE2 (*P* < 0.01) compared with the LV-Mock-treated CUMS group (Figure 6b).

### The administration of meloxicam suppressed the CUMS-induced increase of COX2 expression

**Figure 7:**
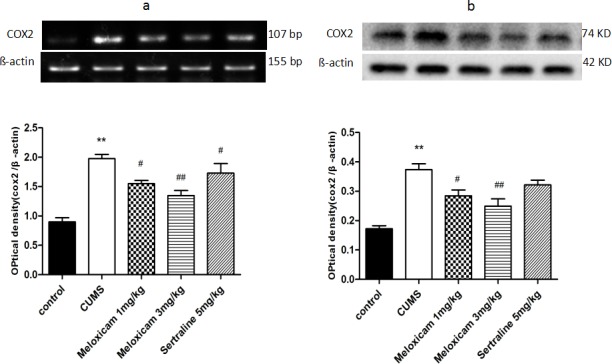
The effects of meloxicam treatment on mRNA and protein expression levels of COX2 in the hippocampus of CUMS rats **a**. Changes in the mRNA expression of COX2 in the hippocampus of CUMS rats after treatment with meloxicam (1 and 3mg/kg, i.g.) or sertraline(5mg/kg, i.g.). **b**. The protein levels of COX2 in the hippocampus of CUMS rats after treatment with meloxicam (1 and 3mg/kg, i.g.) or sertraline(5mg/kg, i.g.). Each value represents the mean ± SD using a one-way ANOVA with a Bonferroni correction. **P < 0.01 vs control group. ^#^ and ^##^ *P* < 0.05 and < 0.01 vs the CUMS group. *n* = 10.

The mRNA and protein expression of COX2 in the hippocampus were measured to observe whether COX2 contributes to CUMS-induced cognitive deficits. As shown in Figure 7, CUMS significantly incresed the mRNA and protein expressions of COX2 in the hippocampus of the CUMS control group (*P* < 0.01 and *P* < 0.01, respectively). Inhibition of COX2 activity with meloxicam (1 or 3 mg/kg) or administration of sertraline (5 mg/kg) led to a significant reduction in the mRNA levels of COX2 (*P* < 0.05, *P* < 0.01 and *P* < 0.05 respectively) compared with CUMS group. COX2 protein levels decreased in meloxicam 1 mg/kg CUMS group (*P* < 0.05), and meloxicam 3 mg/kg CUMS group (*P* < 0.01).

### Inhibition of the COX2 activity enhanced synaptic plasticity related proteins expression in the hippocampus of CUMS-treated rats

**Figure 8:**
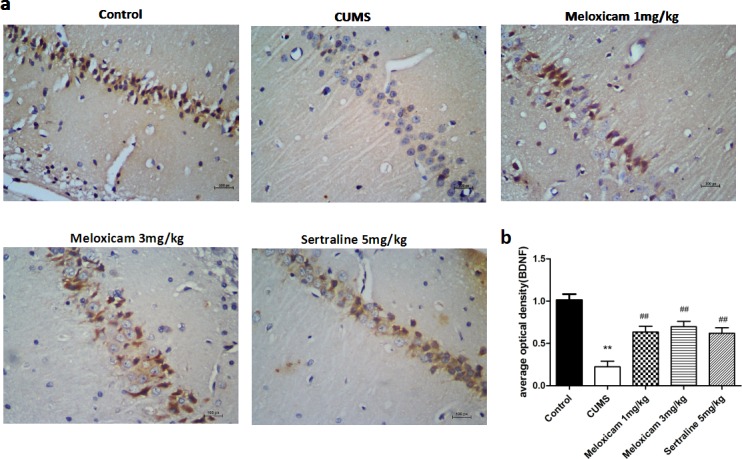
The expression of BDNF protein in the hippocampus of CUMS rats upon the activities of COX2 **a**. Representative immunohistochemical staining for BDNF positive areas in the hippocampal brain sections from CUMS rats after meloxicam (1 and 3mg/kg, i.g.) or sertraline (5mg/kg, i.g.) treatment. **b**. Densitometric analyses of the immunoreactivity to the BDNF antibodies from the previous panel Figure 8 (a). Positive cells are represented as browns spots. Scale bar = 100 um. The level of staining density was quantified by Image-ProPlus 6.0 and presented as the mean ± SD using a one-way ANOVA with a Bonferroni correction. **P < 0.01 vs control group. ^#^ and ^##^*P* < 0.05 and < 0.01 vs the CUMS group. *n* = 10.

BDNF is the protein relate to the synaptic plasticity which was quantified to explore whether the activities of COX2 were related to the learning and memory function in CUMS-treated rats is due to its effect on synaptic plasticity. As shown in Figure 8, compared with control group, BDNF staining intensity was significantly decreased in the cytoplasm of neurons from the CMC-Na-treated CUMS groups (*P* < 0.01). The administration of 1 mg/kg (*P* < 0.01), 3 mg/kg (*P* < 0.01) of meloxicam and 5 mg/kg sertraline (*P* < 0.01) significantly attenuated the reductions.

### Effects of COX2 RNAi and overexpression on the levels of COX2, prostaglandins receptor 3 (EP3), cyclic adenosine monophosphate (cAMP), protein kinase A IIα (PKAIIα) reg, phosphorylated cAMP response element binding protein (p-CREB)/CREB and BDNF in the hippocampus of CUMS-treated rats

**Figure 9:**
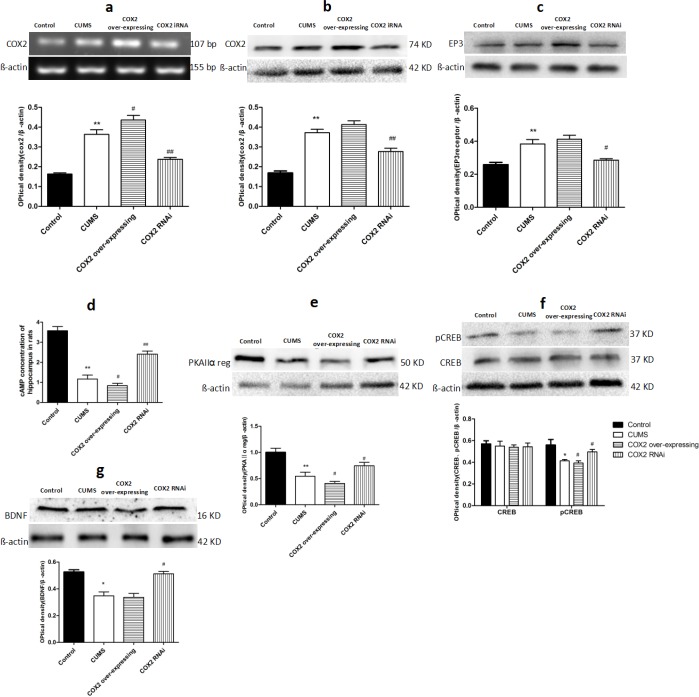
Effects of COX2 RNAi and overexpression on the levels of COX2, EP3, cAMP, PKAIIα reg, p-CREB/CREB, and BDNF in the hippocampus of CUMS-treated rats **a.** Changes in the mRNA expression of COX2 in the hippocampus of CUMS rats after treatment with LV-Mock lentivirus, COX2 over-expressing or LV-si-COX2lentivirus (15 ul, 10 ^8^ TU/ml, intracerebroventricular injection, respectively). The relative protein expressions of COX2 **b**., EP3 **c**., the content of cAMP **d**., the protien level of PKAIIα reg **e**., phosphorylated and total forms of CREB **f**., BDNF **g**. in the hippocampus of CUMS rats after treatment with LV-Mock lentivirus, COX2 over-expressing or LV-si-COX2 lentivirus (15 ul, 10 ^8^ TU/ml, intracerebroventricular injection, respectively). Each value represents the mean ± SD using a one-way ANOVA with a Bonferroni correction. **P < 0.01 vs control group. ^#^ and ^##^ *P* < 0.05 and < 0.01 vs the CUMS group. *n* = 10.

Findings above showed there was an elevation in COX2's downstream product PGE2 levels, a reduction of BDNF levels, and impairment of cognitive in CUMS-treated groups (including CMC-Na-, LV-Mock-treated, and COX2 overexpress lentivirus treated CUMS groups). To study the relationship between the COX2 and BDNF, we quantified the COX2 mRNA, proteins levels of COX2, EP3, PKAIIα reg, p-CREB/CREB, BDNF and levels of cAMP after administration of LV-si-COX2 or overexpressed COX2 lentivirus intracerebroventricular injection. As shown in the Figure 9, compared with the naive group, there was a significantly increase in the content of COX2 mRNA (*P* < 0.01), COX2 protein (*P* < 0.01), EP3 protein (*P* < 0.01), and decrease in the levels of cAMP (*P* < 0.01), PKAIIα reg (*P* < 0.01), p-CREB/CREB (*P* < 0.05), and BDNF (*P* < 0.05) in the hippocampus of LV-Mock-treated CUMS group. Compared with LV-Mock-treated CUMS group, COX2 overexpressed lentivirus intraventricular injection deteriorated the reduction of cAMP (*P* < 0.05), PKAIIα reg (*P* < 0.05), and p-CREB/CREB (*P* < 0.05) and elevated the contents of COX2 mRNA (*P* < 0.05). Whereas LV-si-COX2 raised the levels of cAMP (*P* < 0.01), PKAIIα reg (*P* < 0.05), p-CREB/CREB (P < 0.05), BDNF (*P* < 0.05) and decreased the expression of COX2 mRNA (*P* < 0.01), COX2 protein (*P*< 0.01) and EP3 protein (*P* < 0.05).

## DISCUSSION

To reveal the relationships among depression, inflammation and cognitive function we established rat model of depression with CUMS. As a result, CUMS induce decrease in body weight and sucrose intake. CUMS-treated rats exhibited increased escape latency, decreased number of platform crosses in the MWM test, reduced the latency and increased error number in the step-down-type passive avoidance test. Those depressive symptoms and cognitive deficits accompanied with elevated inflammatory levels and COX2 expression which indicated neuroinflammation in hippocampal neurons. The CUMS exposure groups were treated with meloxicam or LV-si-COX2 to inhibit the activation or suppress the expression of enzyme, resulting in the reduction of inflammation response and a better learning performance. Moreover, overexpressed COX2 lentivirus treated group exhibited by increased expression of the inflammatory cytokines and worsen learning performance. In these scenarios, COX2 play an important role in pathophysiological processes of cognitive dysfunction induced by depression.

To respond and adapt to external or internal stimuli, neuroinflammation has an important role in communicating between the immune and central nervous systems [24]. However, pathological conditions lead to an excessive inflammation response that may have a detrimental impact on neuronal. A growing body of evidence supports the hypothesis that depression is also related to the inflammation [25]. COX2 is a key enzyme in inflammation, its role and the mechanisms through which it is involved in the pathophysiology of mental disorder have been confirmed in many studies. Su's study describes polymorphisms in COX2 gene relevant for IFN-α-induced depression and COX2's levels associated with severity of depressive symptoms [26]. Nery treated bipolar disorder patients with celecoxib to inhibit the activity of COX2, and reported that it could improve their depressive symptom [27]. Consistent with these results, the results obtained in our study showed higher gene expression of COX2 and inflammation marker levels in CUMS treated rats, which are associated with increased severity of depressive symptoms. Additionally, inhibit the activities or suppress the expression of COX2 reduced CUMS-induced inflammation response and depressive symptoms; meanwhile, excessive COX2 gene expression increased neuroinflammation and aggravated emotional disturbances.

Addition to confirmed associate with the depression, it also has been reported that COX2 has an important role in the pathophysiology of cognitive impairment in acute or chronic CNS disorders. In Mexican patients with diabetes [28], less exposure to the CG genotype of the c.1-765G>C polymorphism of COX2 was shown to be associated with minor cognitive decline. COX2-specific inhibitors, in treating brain injuries, improve functional outcomes in rat model of traumatic brain injury.

As a critical enzyme in the synthesis of prostaglandins, COX2 is markedly induced in cerebral and hippocampal neurons by immune stimuli. PGE2, a downstream products of COX2, mediates different components of the physiological and pathophysiological reaction via it's receptors [29]. There are four types of prostaglandins receptor. Markedly, the expression of EP3 receptors has been reported in rat hippocampus and with higher affinity than the other three subtypes [30]. There is a large body of data showing that EP3 receptors are a key component in the progression of pathology in neurotoxic processes. In transient focal ischemia models, EP3 knock-out mice exhibited reduced infarction, edema and neurological dysfunctions compared with wild-type mice. However, stimulating EP3 pharmacologically has been shown to increase infarct size in stroke injury [31]. In 5XFAD APP transgenic mice model of Familial AD, deletion of the EP3 receptor blocked induction of pro-inflammatory gene, protein expression, lipid peroxidation and cognitive decline [32]. EP3 receptors are coupled to G proteins through a Gi/o subunit which negatively regulates AC-cAMP signal transduction pathway and cAMP response element-binding protein (CREB) activation. CREB, a transcription factor, is believed to be activated by PKA and plays important roles in learning and memory in the brain [33]. In neurons, the phosphorylation of Ser133 in CREB leads to the expression of neurotrophic factors genes such as BDNF that regulate survival, growth, synaptic plasticity, and long-term memory. Recent developments linking BDNF to a wide array of pathogenesis in depression and its treatment outcomes are highlighted [34]. Animal studies have shown that both acute and chronic stress could result in the reduction of BDNF expression accompanied with neuronopathies in the hippocampus [35]. In addition to relate to depression, increasing amounts of data suggests that BDNF in hippocampus is a key protein involve in hippocampal neuronal plasticity and learning/memory. BDNF can induce long-term potentiation (LTP), which is considered to be the neurophysiological basis for learning and memory. Furthermore, inhibition of BDNF signaling by gene knockout or antisense RNA impairs spatial learning and memory [36].

Although there are lines of evidence point out the relationship between BDNF and depression or cognitive function, but the association between the COX2 and cognitive impairment in depression is not very clear. The results obtained in our study showed an increase in COX2 expression and its downstream products PGE2 level. To explore the mechanism we detected the expression of EP3 receptor, contents of the second messenger cAMP, levelof PKA, the ratio of pCREB/CREB and the expression of BDNF. The results obtained here exhibited stronger inflammation response accompany with higher levels of PGE2 and activation of cAMP/PKA-CREB-BDNF signaling pathway of hippocampus in CUMS-treated rats. In addition to improving the depressive symptoms and learning/memory dysfunction, the administration of meloxicam or LV-si-COX2 via an intracerebroventricular injection induces reduction of inflammatory contents, COX2 expression, PGE2 levels, EP3 expression, and up-regulation of cAMP/PKA-CREB-BDNF signal pathway. Interestingly, rats treated with COX2 overexpressing lentivirus showed behavioral deterioration with increased inflammation, expression of COX2, levels of PGE2, and down regulation of cAMP/PKA-CREB-BDNF signaling pathway.

Taken together, these results are in agreement with the previous reports, furthermore our study highlight the novel functional role that COX2 is appears to be a key part of the pathophysiology of inflammation induced by CUMS, the main mechanism of COX2 involve in mood and learning/memory disorder is to impair the synaptic function and plasticity in hippocampal neuron through inhibition of cAMP/PKA-CREB-BDNF signaling pathway by PGE2-EP3 in hippocampus.

This new information by demonstrating the role of COX2 in pathogenesis of cognitive dysfunction in depression makes it not only a valid pharmacological target, since COX2 inhibitors are already widely used, but most importantly represents a unique therapeutic opportunity with a true disease-modifying potential for the treatment of cognitive impairment in depression.

## MATERIALS AND METHODS

### Animals

The study was carried out on a group of 130 Sprague-Dawley rats (180-200 g, 8 weeks old) that were obtained from the animal experimental center at Chongqing Medical University. Rats that were subjected to CUMS were housed individually while the control rats were housed in groups of five. All animals were housed under a normal light/dark cycle with food and tap water made available *ad libitum*. The experimental protocols were carried out in accordance to the National Institute of Health Guide for the Care and Use of Laboratory Animals (NIH Publications No. 80-23) and were approved by the Animal Care and Use Committee at Chongqing Medical University.

### Experimental design

Our experiment was performed in two main parts. In the first part, meloxicam, a nonsteroidal anti-inflammatory drug that inhibits the activity of COX2, was administrated to the rats subjected to CUMS and the changes in depression-like behaviors, learning and memory functions, levels of inflammatory cytokines, plasticity of hippocampal neurons and the COX2 expression were observed. In the second part, the expression of COX2 of rat hippocampus was down regulated or up regulated via COX2 RNAi lentivirus and COX2-overexpressing lentivirus through intracerebroventricular injections, during exposure to CUMS. The behavioral changes, inflammation response, and COX2-PGE2-EP3 signaling pathway with its downstream cAMP/PKA-CREB-BDNF pathway in hippocampal neurons were further analyzed.

Section one: A total of 90 rats were randomly divided into a control group (*n* = 10) and a CUMS group (*n* = 80). The CUMS-exposed rats with significant differences in sucrose preference test were further subdivided into four subgroups after 6 weeks of exposure, with 10 rats per group, the remaining animals without significant differences in sucrose preference test were not enroll the following test. One group received 0.5% CMC-Na (orally, 2 ml/kg/day for 21 days), two groups received meloxicam for 21 days (gavage, 1 mg/kg/day or 3 mg/kg/day), and the fourth group received sertraline which used as a positive control (gavage, 5 mg/kg/day for 21 days) [23] (Figure 1a.).

Section two: Forty rats were randomly divided into the following four groups: Mock lentivirus group (*n* = 10), Mock lentivirus + CUMS group (*n* = 10), COX2 over-expressing lentivirus + CUMS group (*n* = 10), and COX2 RNAi lentivirus + CUMS group (*n* = 10). Rats were treated with an empty vector, COX2 over-expressing lentivirus, or COX2 RNAi lentivirus through intracerebroventricular injections (15 ul, 10 ^8^ TU/ml), respectively on the fifth, twelfth, and nineteenth day following exposure to CUMS. Behavioral tests were performed two days following the last injection (Figure 1b).

### CUMS paradigm

The chronic unpredictable mild stress procedure was conducted for 42 or 21 days according to a previously described protocol [37], with slight modifications: 24 h cage tilting (45°), 2 h noise (92 dB, 92 Hz), overnight illumination, 5 min cold swimming at 4°C, 24 h wet bedding, 1 min tail pinch (1 cm from the end of the tail), 24 h water and food deprivation, and 5 min thermal environment (45°C). Rats were individually exposed to the stressors in random order once a day. No single stressor was performed consecutively. Control rats were kept in a separate room without being subjected to any stressors.

### Weight change

All rats were weighed on days 0, 21, 42, or 63 during the experiment (Figure 1).

### Sucrose preference test

The anhedonia induced by the CUMS protocol was assessed using the sucrose preference test according to a previous study, with minor modifications [38]. Rats were trained to acclimatize to a 1% (w/v) sucrose solution prior to the test: two bottles of sucrose solution were placed on each cage for 24 h, one bottle of sucrose was placed with water for the subsequent 24 h. Sucrose and water consumption was measured by comparing bottle weights before and after placing the bottles on top of the cage, during the 1 h window after 24 h of fasting.

### Morris water maze

Rats were trained and tested in the MWM to monitor their spatial learning and memory, following a reported method [39]. Rats were given four trials per day for four consecutive days. A different entry site was used for each daily session. During each trial, the rats were introduced into the water where a hidden platform was submerged under the water. If rats failed to reach the platform within 180 sec, they were gently guided to it and allowed to remain for 10 sec on top of the platform. On the 5th day, following the last day of training, rats were introduced into the pool from the entry site where the last training was performed in order to assess retention of the platform location. During this probe trial, the platform was removed from the maze. The latency to find the hidden platform and the number of times crossing the platform were recorded, with a maximum of 180 sec.

### Step-down-type passive avoidance test

The step-down-type passive avoidance test consisted of two sessions according to a previously described method with minor modifications [38]. In the training session, the animal was placed in the box for 3 min and allowed to explore the cage freely. Rats were then exposed to an electric shock (30 V for 5 min) until they stepped onto a rubber platform. After a 24 h interval, rats were placed on the platform and the step-down latency and the number of errors was recorded. The cut-off time in both sessions was 300 s.

### Intracerebroventricular injections

Rats were anesthetized with 4% chloral hydrate and stereotaxically implanted with a stainless steel guide cannula according to a procedure as described by Haley and McCormick [40]. Cannula inserted ventricular catheter coordinates were leftward 1.2 mm and 1 mm posterior to the bregma, 4.5 mm below the dura in the midline, then fixed catheter with dental cement. The 5th, 12th, 19th day after surgery, rats were injected lentivirus (15 ul, 0.5 ul/min, retaining needle 10 min to prevent reverse flow of liquid) through the ventricular catheter.

### Western blotting

Hippocampus tissue (50 mg, *n* = 4) was homogenized in 0.5 ml tissue lysate buffer for protein extraction and centrifuged at 12, 000 × g at 4°C for 15 min. The supernatant was collected and protein concentrations were measured with the bicinchoninic acid (BCA) protein assay kit (Beyotime, China). Western blotting was performed as previously described [40]. Briefly, protein was separated by sodium dodecyl sulphate polyacrylamide gel electrophoresis (SDS-PAGE) and immunoblotted with antibodies against COX2, EP3, p-CREB, CREB, BDNF, PKAIIα reg (1:500; Santa, USA) and ß-actin (1:3000; Sigma, USA) as loading control. The color reaction was visualized by enhanced chemiluminescence (ECL) reagents (Pierce, USA). A Bio-Rad imaging system was used to detect and quantify protein levels.

### Reverse transcription polymerase chain reaction (RT-PCR)

To determine the expression of COX2 mRNA in rat hippocampus, total RNA was extracted from the hippocampus tissue using TRIzol reagent (Takara, Japan). RT-PCR system included 1 μg total RNA, 1 μmol/L oligo (dT), 0.2 mmol/L diethyl-nitrophenyl thiophosphate (dNTPs), 10 U RNase inhibitor, and 4 U ReverTra Ace (Takara, Japan). The reaction consisted of 20 min at 42°C, 5 min at 99°C, and then 5 min at 4°C. Amplification was carried out in 0.2 mmol/L dNTPs, 2 mmol/L magnesium chloride, 1 μmol/L of each primer, and 2.5 U Taq DNA polymerase (Promega, USA), and consisted of the following steps: initial denaturation at 94°C for 4 min, followed by 35 cycles at 94°C for 15 sec, 53.1°C for 15 sec, and 72°C for 40 sec, annealing at 55.5°C for 15 sec, and a final extension at 72°C for 5 min. The primers of COX2 and ß-actin mRNA were purchased from DINGGUO Biotech Incorporated Company (Beijing, China). The COX2 primers were designed using Primer Premier 5.0 (Premier Biosoft International, USA) on the basis of the rat COX2 cDNA sequence in GeneBank. The primer sequences for COX2 were: forward 5′-TGAACACGGACTTGCTCACTTTG-3′ and reverse 5′-AGGCCTTTGCCACTGCTTGTA-3′ (107 bp), and ß-actin with forward 5′-ACGGTCAGGTCATCACTATCG-3′ and reverse 5′-GGCATAGAGGTCTTTACGGATG-3′ (155 bp). The amplified products were separated by 2% agarose gel electrophoresis, and were visualized by ethidium bromide staining. The optical density of COX2 was determined with Quantity One software (Bio-Rad), and expressed as the ratio against ß-actin.

### Biochemical analysis of hippocampal tissues

Rat hippocampus tissues (*n* = 4) were homogenized with histological saline (weight/volume, 1:9). Subsequently, the homogenates were centrifuged at 14, 000× g for 20 min, and the supernatant was collected to measure the levels of cAMP and inflammation cytokines by using rat cAMP (R&D Systems China shanghai, China), PGE2 (TaKaRa Japan), TNF-α and IL-6 enzyme-linked immunosorbent assay kits (Huamei Bioengineering Institute, China).

### Immunohistochemistry

Immunostaining was performed to investigate the expression of BDNF in the rat hippocampus as reported previously [39]. Briefly, sections were dewaxed and rehydrated in ethanol with decreasing concentration, high pressure antigen retrieval was performed in citrate buffer for 10 min prior to peroxidase quenching with 3% hydrogen peroxide in phosphate-buffered saline (PBS) for 10 min. Slides were washed with PBS for three times (5 min each) and were pre-incubated in 1% serum for 30 min at room temperature. Then, slides were incubated with a rabbit polyclonal antibody (BDNF 1:50, Santa Cruz, USA.) overnight at 4°C. Sections were subsequently incubated with biotinylated secondary antibody (1:400, Bio-lab, China) for 30 min at 37°C, then with streptavidin for 20 min, and were washed with PBS for another three times (5 min each). Antigens were visualized with DAB solution. Sections were counterstained with hematoxylin and then examined under light microscopy. The Integral Optical Density (IOD) was analyzed using the image-pro plus 6.0 software.

### Statistical analysis

All experimental data were expressed as mean ± standard deviation (SD). Data for the MWM were assessed by repeated measures analysis of variance (ANOVA). Data regarding sucrose preference, step-down-type passive avoidance test, changes in COX2 expression, and levels of protein and inflammatory mediators were determined by one-way ANOVA followed by the least significant difference test (*P* < 0.05) using the Statistics Package for Social Sciences program version 11.5.

## Abbreviations

BCA: bicinchoninic acid
BDNF: brain-derived neurotrophic factor
CREB: cAMP response element binding protein
cAMP: cyclic adenosine monophosphate
COX2: Cyclooxygenase 2
CUMS: chronic unpredictable mild stress
dNTPs: diethyl-nitrophenyl thiophosphate
ECL: enhanced chemiluminescence
IL: Interleukin
IFN-α: interferon alpha
LV: lentivirus
LTP: long-term potentiation
mRNAs: messenger RNAs
MWM: Morris water maze
NSAIDs: Non-steroidal anti-inflammatory drugs
PGE2: prostaglandin E2
EP3: prostaglandins receptor 3
PKA: protein kinase A
PBS: phosphate-buffered saline
RNA: interfere-RNA
CMC-Na: sodium carboxymethyl cellulose solution
SPT: Sucrose preference test
SDS-PAGE: sodium dodecyl sulphate polyacrylamide gel electrophoresis
TNF-α: tumor necrosis factor alpha
